# Progress in Electromagnetic Wave Absorption of Multifunctional Structured Metamaterials

**DOI:** 10.3390/polym17182559

**Published:** 2025-09-22

**Authors:** Zhuo Lu, Luwei Liu, Zhou Chen, Changxian Wang, Xiaolei Zhu, Xiaofeng Lu, Hui Yuan, Hao Huang

**Affiliations:** 1 School of Mechanical and Power Engineering, Nanjing Tech University, Nanjing 211800, China; luzhuo2023@163.com (Z.L.); lwliu2012@163.com (L.L.); zhuxiaolei@njtech.edu.cn (X.Z.); xflu@njtech.edu.cn (X.L.); yzx9807@sina.com (H.Y.); 2 School of Mechanics and Construction Engineering, Jinan University, Guangzhou 510632, China; wangcx@jnu.edu.cn; 3 National Innovation Institute of Defense Technology, Academy of Military Sciences, Beijing 100071, China; huanghao@nudt.edu.cn

**Keywords:** multifunctional metamaterials, electromagnetic wave absorption, broadband absorption, impedance matching, mechanical properties, multiphysics coupling

## Abstract

This review summarizes recent advances in multifunctional metamaterials (MF-MMs) for electromagnetic (EM) wave absorption. MF-MMs overcome the key limitations of conventional absorbers—such as narrow bandwidth, limited functionality, and poor environmental adaptability—offering enhanced protection against EM security threats in radar, aerospace, and defense applications. This review focuses on an integrated structure-material-function co-design strategy, highlighting advances in three-dimensional (3D) lattice architectures, composite laminates, conformal geometries, bio-inspired topologies, and metasurfaces. When synergized with multicomponent composites, these structural innovations enable the co-regulation of impedance matching and EM loss mechanisms (dielectric, magnetic, and resistive dissipation), thereby achieving broadband absorption and enhanced multifunctionality. Key findings demonstrate that 3D lattice structures enhance mechanical load-bearing capacity by up to 935% while enabling low-frequency broadband absorption. Composite laminates achieve breakthroughs in ultra-broadband coverage (1.26–40 GHz), subwavelength thickness (<5 mm), and high flexural strength (>23 MPa). Bio-inspired topologies provide wide-incident-angle absorption with bandwidths up to 31.64 GHz. Metasurfaces facilitate multiphysics functional integration. Despite the significant potential of MF-MMs in resolving broadband stealth and multifunctional synergy challenges via EM wave absorption, their practical application is constrained by several limitations: limited dynamic tunability, incomplete multiphysics coupling mechanisms, insufficient adaptability to extreme environments, and difficulties in scalable manufacturing and reliability assurance. Future research should prioritize intelligent dynamic response, deeper integration of multiphysics functionalities, and performance optimization under extreme conditions.

## 1. Introduction

The increasing need for electromagnetic (EM) stealth in radar, aerospace, and military applications underscores the profound impact of EM radiation on modern technology. However, EM waves induce reflection, scattering, and interference, degrading communication integrity and posing threats to electronic systems. To address these challenges, MMs have emerged. By absorbing incident EM waves and converting them into thermal energy [1], MF-MMs effectively suppress reflection and scattering, thereby mitigating EM interference. Concurrently, advancements in stealth technology have established MMs as critical components with broad application prospects. While conventional absorbers partially reduce EM reflection and scattering, they suffer from inherent limitations-such as large thickness [2], high density [3], and narrow operational bandwidth. In contrast, MF-MMs demonstrate distinct advantages in these aspects [4]. With continued technological progress, MF-MMs are positioned to supersede traditional absorbers across multiple domains, emerging as the dominant solution for EM wave absorption.

MMs dissipate EM energy through dielectric polarization losses, magnetic hysteresis losses, and multimodal resonance mechanisms [5], enabling extensive research and applications in EM protection for military/civil infrastructure. Nevertheless, conventional absorbers typically achieve effective absorption (reflection loss ≤ −10 dB) only within discrete frequency bands [6], constrained by absorber composition, layer thickness, and bulk density. Although compositional modifications—such as optimizing absorber selection, microstructure, and doping—can improve attenuation, monolithic systems usually provide adequate absorption only within narrow microwave bands, lacking the integration of broadband strong absorption with multifunctionality. MF-MMs overcome these limitations through architectural design of polymer-based structures [7], enhancing impedance matching at air-material interfaces to maximize EM wave penetration. Structure-material synergy further enables ultra-broadband absorption [8], significant lightweighting, and customizable multifunctionality [9]. To advance strong wideband absorption and multifunctional performance [10], this work comprehensively focuses on four key aspects: (1) contemporary MF-MM topological configurations; (2) polymer composite material systems; (3) EM absorption enhancement mechanisms; and (4) practical engineering applications [11].

## 2. Mechanisms of Electromagnetic Wave Absorption in Multifunctional Metamaterials

**Figure 1:**
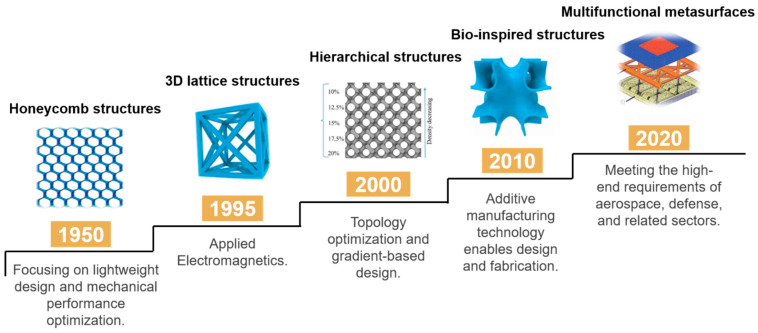
Key milestones in the development of multifunctional metamaterials.

MF-MMs are artificially engineered material systems characterized by specific, often periodic or aperiodic, geometric arrangements [12,13]. Their evolution—from homogeneous media in the 1940s, to periodic metamaterials in the early 21st century, and now to disordered/composite metamaterials [14]—reflects continuous exploration and innovation in materials science [15]. As shown in Figure 1, the EM wave absorption mechanism primarily relies on achieving effective impedance matching between the structure and constituent materials [16], enabling efficient and broadband EM wave absorption. Through precise structural design, metamaterials can modulate the interaction with EM waves, inducing strong resonances within targeted frequency bands. This facilitates the conversion of incident wave energy into heat or other dissipated energy forms, thereby minimizing reflection and transmission [17]. Furthermore, the synergistic integration of tailored structures and composite materials enables multifunctionality [18], including broadband operation, lightweight properties, and tailorable performance [19].

### 2.1. Electromagnetic Wave Absorption Efficiency

EM wave absorption is typically quantified using Reflection Loss (RL) in decibels (dB), calculated from the material’s reflection, transmission, and absorption properties [20]. When an EM wave impinges on a metamaterial surface, the energy partitions into three components: a reflected portion, an absorbed portion, and a transmitted portion. The absorption efficiency (*A*) represents the ratio of absorbed energy to total incident energy. Higher absorption corresponds to stronger EM wave dissipation within the material [21].

As a core metric for evaluating the EM wave absorption capabilities of metamaterials, the absorption coefficient critically determines the practical deployment effectiveness of metamaterials. This parameter depends on three interdependent factors: the intrinsic EM properties of constituent materials, the structural configuration, and the operational EM parameters of the application environment. By optimizing these variables in parameter space and refining the geometry of metastructures, researchers can significantly enhance EM absorption efficiency and expand the operational bandwidth. For EM waves incident normal to the metamaterial interface, Absorption (*A*) is governed by Equation (1) [22].

(1) A=1−R−T

In Equation (1), *R* represents the power reflection coefficient of the metamaterial absorber, while *T* denotes its power transmission coefficient. Both parameters are derived from complex S-parameters through the computational relationships expressed in Equations (2) and (3) [23].

(2) $${R=\left|S_{11}\right|}^{2}$$

(3) $$T={\left|S_{21}\right|}^{2}$$

Within the context of EM wave interaction, $S_{11}$ denotes the reflection coefficient, while $S_{21}$ represents the transmission coefficient. Consequently, the wave absorption (*A*) of a metamaterial is defined by the equation *A* = 1 − ${\left|S_{11}\right|}^{2}$ − ${\left|S_{21}\right|}^{2}$. This formulation explicitly describes the fundamental absorption mechanism in metamaterials. To maximize this efficiency, the reflectance and transmittance of the incident wave must be minimized through tailored structural design [24]. Table 1 summarizes the key metrics for evaluating the EM wave absorption performance of such metamaterials.

### 2.2. Electromagnetic Wave Absorption Mechanism

**Figure 2:**
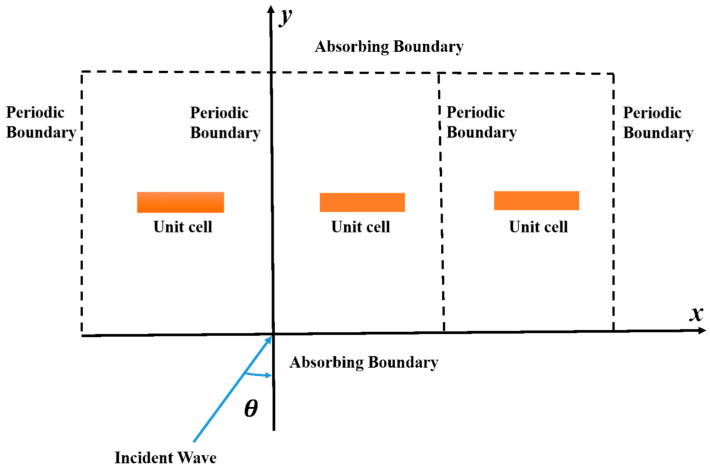
Schematic of Electromagnetic Wave Absorption Mechanisms in Metamaterials.

Metamaterials manipulate EM wave absorption by engineering unit cells into periodic or aperiodic configurations and tailoring surface textures. This structural design dynamically modulates the composite’s effective permittivity and permeability, enabling precise control of impedance matching and EM dissipation mechanisms. Consequently, incident waves are guided into the material’s interior for efficient energy conversion, as depicted in Figure 2. Key loss mechanisms include quarter-wavelength (*λ*/4) resonance, edge diffraction effects [25], multi-reflection and absorption [26], and polarization/magnetic loss attenuation [27]. When EM waves interact with surface protrusions, anomalous reflection and surface wave coupling redirect propagation trajectories and scattering patterns, suppressing specular reflection. Concurrently, scattering/diffraction phenomena at structural edges enhance destructive interference losses through EM energy redistribution.

### 2.3. Electromagnetic Parameters

The EM response of metamaterials is primarily dictated by their inherent constitutive parameters, among which the complex permittivity (*ε*) and complex permeability (*μ*) are paramount. These frequency-dependent complex quantities comprise real components (*ε*′, *μ*′) that quantify the material’s dielectric and magnetic polarization under EM excitation, while imaginary components (*ε*″, *μ*″) characterize intrinsic energy dissipation through dielectric and magnetic losses [28]. This fundamental relationship between EM response and material polarization/dissipation mechanisms is formally captured by Equations (4) and (5).

(4) $$\varepsilon =\varepsilon^{\prime }-j\varepsilon^{\prime \prime }$$

(5) $$\mu =\mu^{\prime }-j\mu^{\prime \prime }$$

The real part of complex permittivity (*ε*′) quantifies the material’s capacitive energy storage, while its imaginary component (*ε*″) characterizes dielectric loss arising from polarization relaxation processes within the material. This electrical dissipation scales proportionally with (*ε*″) magnitude. Similarly, the real permeability component (*μ*′) governs inductive energy storage, and the imaginary term (*μ*″) represents magnetic loss attributed to magnetic dipole reorientation dynamics, with loss magnitude proportional to *μ*″.

To achieve impedance-matched EM wave penetration with minimal reflection, precise tuning of these constitutive parameters is essential [29]. Furthermore, the material’s loss behavior is comprehensively described by the dielectric loss tangent $\left(\tan\delta_{\varepsilon }\right)$ and magnetic loss tangent $(\tan\delta_{\mu })$, which are dimensionless quantities derived from the imaginary to real ratio of complex permittivity and permeability, respectively. These critical metrics are mathematically expressed as Equations (6) and (7).

(6) $$\tan\delta_{\varepsilon }=\frac{\varepsilon^{\prime \prime }}{\varepsilon^{\prime }}$$

(7) $$\tan\delta_{\mu }=\frac{\mu^{\prime \prime }}{\mu^{\prime }}\;$$

The dielectric loss tangent ($\tan\delta_{\varepsilon }$) quantifies the material’s dielectric loss performance, with dissipation enhancement directly proportional to increases in *ε*″. Similarly, the magnetic loss tangent ($\tan\delta_{\mu }$) characterizes magnetic loss capability, exhibiting proportional enhancement with rising *μ*″ values [30]. Based on the relative dominance of these loss tangents, materials are classified as either predominantly magnetic-loss type (dominated by $\tan\delta_{\mu }$) or dielectric loss type (dominated by $\tan\delta_{\varepsilon }$) absorbers [31].

### 2.4. Impedance Matching

In the field of EM wave absorption, structural metamaterials exhibit outstanding performance due to their unique designs, among which the impedance matching principle serves as the core element for achieving efficient wave absorption. Theoretically, in classical electromagnetism, the energy of EM waves splits into three components—reflection, transmission, and absorption—when interacting with MMs. Impedance matching is designed to minimize reflection and transmission, thereby promoting the absorption of more EM wave energy by MMs and ultimately enhancing wave absorption efficiency [32]. The reflection coefficient *Γ* is used to quantify the impedance matching degree of metamaterials and can be expressed by Equations (8) and (9) [33]:

(8) $$\Gamma =\frac{Z-Z_{0}}{Z+Z_{0}}$$

(9) $$Z_{0}=\sqrt{\frac{\mu_{0}}{\varepsilon_{0}}},\;Z=\sqrt{\frac{\mu }{\varepsilon }}$$

where $Z_{0}\;$ denotes the characteristic impedance of free space, governed by the permeability $\mu_{0}\;$ and permittivity $\varepsilon_{0}$ of vacuum, and *Z* represents the characteristic impedance of the material, which depends on its relative permeability μ and relative permittivity ε. Under ideal conditions, when the characteristic impedance *Z* of the material equals the free-space characteristic impedance $Z_{0}$ (i.e., *Z* = $Z_{0}$), the reflection coefficient *Γ* approaches zero. In this case, electromagnetic waves can penetrate into the material without obstruction, achieving perfect impedance matching and realizing reflectionless transmission—laying the foundation for the efficient absorption of electromagnetic waves [34]. However, most structural MMs have finite-boundary structures in practical applications—differing significantly from the infinite homogeneous medium model underlying classical formulas. Thus, when designing impedance matching for structural MMs, the characteristic impedance *Z* in Equation (8) must be replaced with the structural input impedance $Z_{in}$. This equivalent impedance is not solely determined by the intrinsic properties of the material, but rather an equivalent impedance that integrates multiple factors, including the electromagnetic response of metamaterial units, the overall structural thickness, the interlayer coupling effect, and the properties of the substrate material. This modification also serves as a core prerequisite that must be followed when designing impedance matching for structural metamaterials.

Compared with natural materials, which have limitations in impedance regulation due to their intrinsic properties, structural MMs can break through such constraints via artificial design. Impedance matching in structural MMs is mainly achieved through two approaches: the first is the synergistic regulation of equivalent electromagnetic parameters, which makes the equivalent relative permeability $\mu_{eff}$ and permittivity $\varepsilon_{eff}$ approach equality within the target frequency band, thereby driving the structural input impedance to be close to the characteristic impedance in free space [35]. If parameter imbalance occurs due to multifunctional integration, the parameters can alternatively be adjusted by doping magnetic particles, or $\mu_{eff}$ can be optimized using the unit resonance effect. Second, structural optimization is employed to achieve precise impedance matching. This includes constructing gradient-index structures to create a smooth impedance transition for broad-bandwidth performance, designing multi-functional resonant units that unify impedance manipulation with functional integration, and employing metasurface-bulk hybrid configurations in which the metasurface acts as an ultrathin matching layer while the bulk material provides core absorption and other synergistic functionalities.

### 2.5. Multifunctional

MF-MMs are architected materials that achieve unprecedented physical properties through subwavelength structural design [36]. Unlike conventional metamaterials, these systems enable simultaneous modulation of EM, mechanical, and thermal properties via strategic arrangement of tailored unit cells. Recent advancements in additive manufacturing and computational design have driven metastructures to revolutionary applications in stealth technology, flexible electronics, and advanced aerospace thermal management.

Notably, EM functionality permits tailored stealth characteristics through precisely engineered configurations [37], serving as a critical enabler for military applications. Mechanical performance manifests exceptional attributes: auxetic re-entrant configurations exhibit superior shear/compressive strength [38], while pyramidal lattice structures demonstrate enhanced vibration isolation [39], enabling aerospace and civil engineering implementations. Thermal management capabilities emerge from optimized metastructures that provide simultaneous thermal insulation and dissipation pathways, addressing critical needs in electronics cooling and high-temperature protection [40]. Concurrently, photonic bandgap engineering through rationally designed meta-atoms enables frequency-selective optical confinement [41], advancing multifunctional material paradigms [42]. Material stability depends on compositional selection and geometric organization. For instance, intermetallic compounds offer exceptional corrosion resistance and thermal resilience owing to their unique bonding configurations [43]. As multifunctional platforms integrating EM, mechanical, and thermal responses [44], these materials exhibit transformative potential across disciplines, with prospects continually expanding through innovations in composite science and digital fabrication technologies.

## 3. Research Progress in Multifunctional Metamaterials for Electromagnetic Wave Absorption

Amidst the rapidly evolving technological landscape, increasingly complex EM environments have heightened demand for high-performance EM wave absorbers. Metamaterials leverage their tailorable architectures to enable precise EM wave manipulation, demonstrating exceptional potential for EM absorption applications. Recent advances in multifunctional metamaterial absorbers have yielded diverse structural archetypes, which synergistically optimize absorption performance and auxiliary performance metrics via innovative design paradigms. These developments provide critical foundations for multidisciplinary applications. Selection of optimal configurations depends on specific operational requirements, with multifunctional EM absorbers systematically categorized according to structural hierarchy (micro/macroscale design principles) as summarized in Table 2.

### 3.1. Three-Dimensional Lattice Structures

3D lattice structures comprise periodic arrangements of unit cells forming porous architectures characterized by low mass density, high porosity, and tailorable geometry. These systems simultaneously achieve enhanced mechanical properties (e.g., specific strength/stiffness) and functional capabilities such as EM wave manipulation [45]. For EM absorption applications, synergistic architectural-material co-design enables efficient broadband dissipation, establishing 3D lattices as a significant research focus in MF-MMs. Predominant configurations include auxetic honeycombs, truss-based frameworks, and crystalline-inspired lattice topologies.

#### 3.1.1. Honeycomb Structures

**Figure 3:**
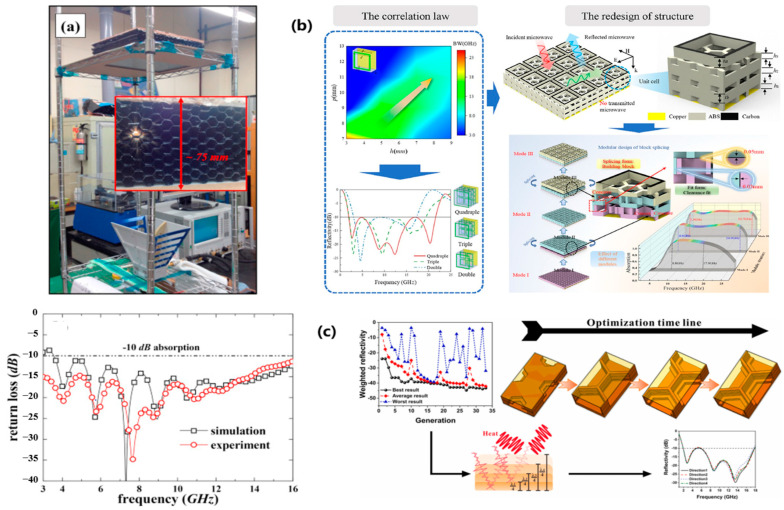
Honeycomb structures: (**a**) Non-magnetic honeycomb configuration; (**b**) 3D-printed carbon-based honeycomb structure; (**c**) Dielectric-magnetic nanocomposite-integrated honeycomb architecture [47,49,51]. Reproduced with permission from Composites Part B: Engineering and Composites Science and Technology.

Honeycomb structures feature periodically arranged hollow cells with 60–95% porosity, conferring exceptional specific strength and lightweight characteristics while enabling tailored EM wave modulation [46]. Synergistic material-structural co-designs address the inherent limitations of conventional absorbers. As shown in Figure 3a, Choi et al. [47,48] demonstrated a non-magnetic configuration achieving >90% absorption across 3–16 GHz, validating structural control for broadband performance. As shown in Figure 3b, Wang et al. [49] realized 2.7–25.2 GHz absorption at 0.52 g/cm^2^ areal density via 3D-printed honeycombs, while Kim et al. [50] maintained effective absorption within 5.8–18 GHz—both exhibiting polarization-insensitive stability. Notably, as shown in Figure 3c, Huang et al. [51] integrated dielectric-magnetic nanocomposites to achieve ultra-broadband absorption (1.92–17.6 GHz) while ensuring mechanical robustness—a tensile strength of 108.6 MPa and a bending load of 0.873 kN. This design successfully reconciles broadband absorption with structural integrity.

This study elucidates the distinctive EM absorption mechanisms inherent to honeycomb structures, encompassing three key aspects: (1) enhanced dissipation through multiple scattering within periodic cavities; (2) dielectric and magnetic losses generated by cell walls; and (3) impedance gradient matching. These synergistic mechanisms enable strategic applications in defense stealth systems and aerospace technologies. Despite persistent challenges in terahertz band extension and environmental stability, the exceptional design flexibility and functional integrability of honeycomb architectures ensure their continued prominence in multifunctional absorber development.

#### 3.1.2. Truss Structures

**Figure 4:**
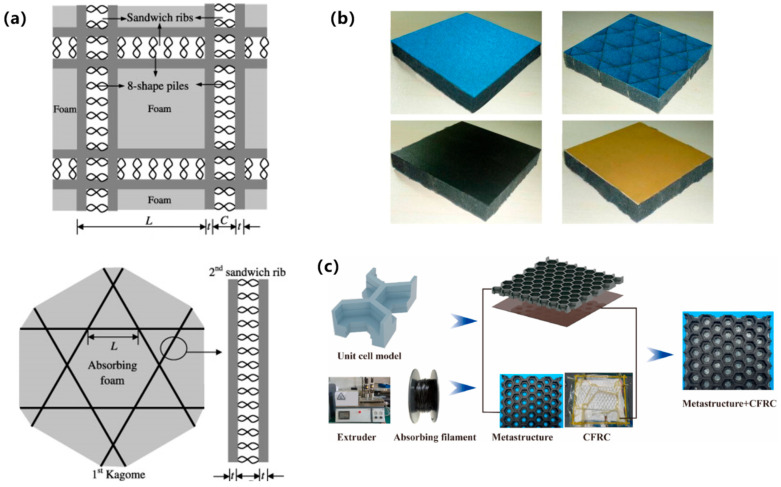
Truss structures: (**a**) Glass fiber-reinforced radar-absorbing truss structure; (**b**) Kagome lattice absorber; (**c**) 3D-printed carbon fiber/nanotube composite truss architecture [53,54,55]. Reproduced with permission from Composites Part B: Engineering and Materials & Design and Composites Science and Technology.

Truss structures feature periodic rod assemblies interconnected at nodal points, forming lattice topologies whose tunable geometric parameters enable precise EM wave modulation [52]. These architectures optimize impedance matching through graded porous designs while leveraging rod arrangements to construct resonant meta-atoms, achieving tunable EM losses from microwave to terahertz frequencies. As shown in Figure 4a, Zheng et al. [53] integrated radar-absorbing foam within glass fiber-reinforced truss frameworks, demonstrating simultaneous EM dissipation and mechanical energy absorption with ultralight characteristics. As shown in Figure 4b, Yang et al. [54] engineered a Kagome lattice achieving broadband absorption (4–18 GHz, RL < −10 dB), confirming electromagnetic-mechanical co-design capabilities. As shown in Figure 4c, Lei et al. [55] fabricated a 3D-printed carbon fiber/nanotube composite truss exhibiting dual-band absorption (2–6.8 GHz, 10.4–40 GHz) with 1.41 KN load-bearing capacity, exemplifying integrated structure-material-function optimization.

Research reveals truss absorption mechanisms include: multi-scattering enhancement via periodic meshes, interfacial polarization at rod surfaces, and synergistic dielectric/magnetic losses [56]. These properties enable strategic deployment in aerospace EM protection systems. Despite challenges in multi-octave bandwidth realization and environmental stability, truss structures’ intrinsic lightweight, high-strength, and functionally scalable advantages will continue to drive multifunctional absorber engineering applications.

#### 3.1.3. Other Periodic Lattice Structures

**Figure 5:**
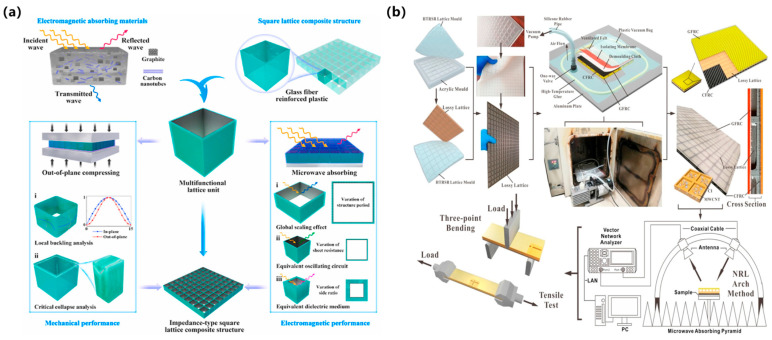
Lattice architectures: (**a**) GFRP square lattice; (**b**) Nanocomposite lattice architecture [58,59]. Reproduced with permission from Composites Science and Technology and Carbon.

Lattice architectures enable unprecedented EM modulation through periodic topological configurations and programmable geometric parameters. By precisely engineering unit cells and optimizing material compositions, these structures achieve broadband efficient absorption spanning microwave to terahertz frequencies [57]. As shown in Figure 5a, Zhang et al. [58] demonstrated a Glass fiber-reinforced polymer (GFRP) square lattice maintaining 5.4–18 GHz absorption bandwidth under 40 MPa compressive stress. As shown in Figure 5b, Huang et al. [59] achieved 3.42–19.73 GHz broadband absorption with their nanocomposite lattice structure, demonstrating 167.35 MPa compressive strength and 5.45% fracture strain, thus overcoming conventional absorbers’ mechanical limitations. Shin et al. [60] attained −29.2 dB reflection loss in Ku-band (12.87–17.78 GHz) with their carbon nanotube-reinforced lattice while matching commercial composites’ mechanical performance. Cui et al. [61] demonstrated >90% absorption across 6.2–22.2 GHz using flexible lattice structures exhibiting exceptional environmental stability. Pang et al. [62] exemplified additive manufacturing capabilities through carbon fiber-assisted lattice fabrication achieving 8.7–19.2 GHz broadband absorption at 2.7 mm thickness.

These advances reveal core modulation mechanisms: Enhanced scattering due to periodicity, impedance matching through cell-size optimization, and composite-driven dielectric/magnetic loss synergy. Despite progress, specific periodic lattice structures exhibit notable practical gaps: crystalline-inspired lattices, for example, achieve superior compressive strength (>160 MPa) but require multi-objective optimization of lattice unit size and material doping—currently a time-consuming process with no standardized workflows. Scalable manufacturing also remains challenging for truss-based lattices, as additive manufacturing of high-aspect-ratio rods leads to >15% dimensional deviation. Future research must establish comparative benchmarks for lattice performance, such as ‘load-bearing efficiency vs. absorption bandwidth,’ to prioritize structural designs for specific aerospace subfields (e.g., UAV skins vs. satellite components).

Future research on 3D lattice structures will prioritize three critical pathways: intelligent adaptive architectures utilizing phase-change materials or reconfigurable geometries for dynamic absorption tuning; multi-physics synergistic optimization balancing EM, mechanical, and thermal performance; and cross-scale manufacturing innovations enabling large-scale fabrication of complex structures to bridge laboratory prototypes and engineering applications. The EM absorption characteristics of these lattice configurations are systematically compared in Table 3.

### 3.2. Composite Layered Structures

Composite layered structures achieve graded EM performance optimization through stratified arrangement of functional layers and interfacial engineering. The core innovation resides in overcoming conventional thickness-bandwidth trade-offs via strategic interlayer coupling design. Structural taxonomy encompasses multilayer, graded-index, and sandwich configurations based on compositional architecture.

#### 3.2.1. Multilayer Structures

**Figure 6:**
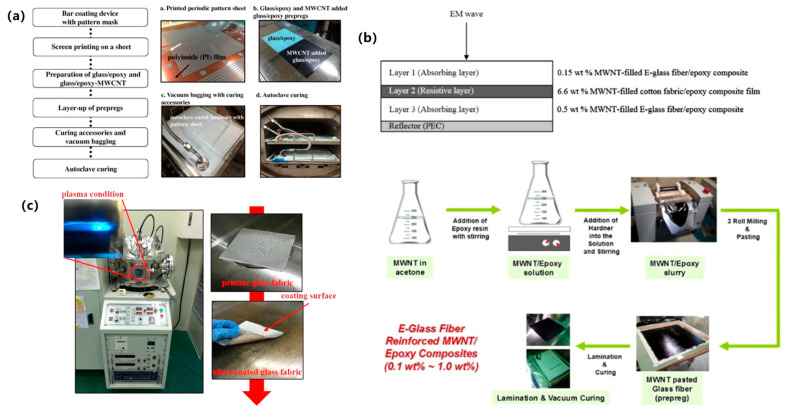
Multilayer structures: (**a**) Hybrid circuit-analog dual-plate structure; (**b**) Three-layer carbon nanotube-dielectric composite structure; (**c**) Conductive polymer EM bandgap (EBG) multilayer architecture [63,64,65]. Reproduced with permission from the Composite Structures and Composites Science and Technology.

Multilayer structures realize the synergistic optimization of “ultra-thin, broadband, and mechanically robust” properties through innovative interlayer coupling engineering. As shown in Figure 6a, Choi et al. [63] engineered a hybrid circuit-analog dual-plate structure attaining <−10 dB reflection loss across X-band (8.2–12.4 GHz) at 2.37 mm thickness. As shown in Figure 6b, They further developed a three-layer composite synergizing multi-walled carbon nanotube resistive layers with dielectric absorbers, extending bandwidth to 4.7–13.7 GHz with −22.3 dB peak absorption [64]. As shown in Figure 6c, Lee et al. [65] designed a conductive polymer EM bandgap (EBG) structure maintaining >90% X-band absorption with <10% mechanical property degradation, while Nam et al. [66] implemented dispersion-stable carbon nanomaterials eliminating conventional processing-induced mechanical compromises.

These designs rely on three core mechanisms: (1) impedance matching layers to extend EM propagation paths; (2) multi-mechanism loss layers to enhance energy conversion; and (3) reinforcement interfaces to preserve structural integrity. Multilayer architectures enable strategic implementations in stealth aircraft skins and satellite radomes. Their inherent design flexibility and process compatibility continue driving EM stealth toward ultra-thin, intelligent multifunctional systems.

#### 3.2.2. Gradient Structures

**Figure 7:**
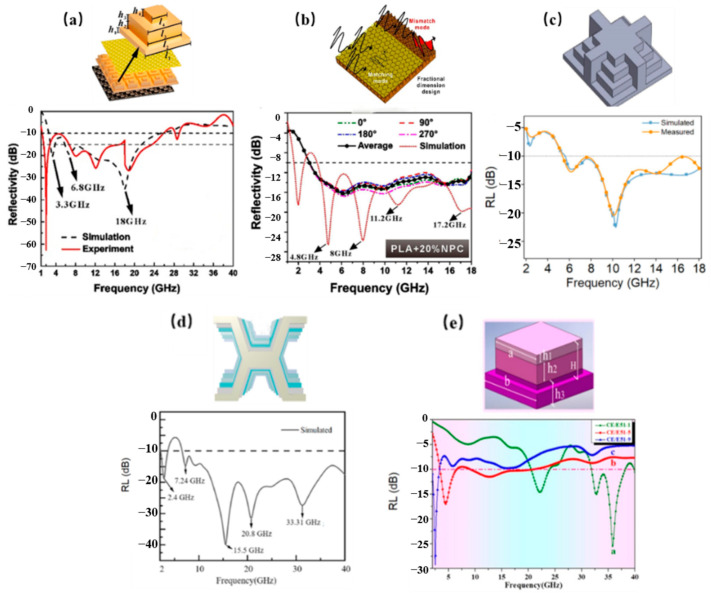
Gradient structures: (**a**) Quartz fiber/nanotube composite gradient structure; (**b**) 3D-printed Kelvin honeycomb gradient architecture; (**c**) Whale-algorithm-optimized plate-lattice gradient structure; (**d**) Short carbon fiber-reinforced gradient configuration; (**e**) Ultrathin (5 mm) gradient-adaptive structure [67,68,69,70,71]. Reproduced with permission from Composites Science and Technology and Composites Part A: Applied Science and Manufacturing and the Chemical Engineering Journal and Composites Communications and Composites Part A: Applied Science and Manufacturing.

Gradient structures enable graded EM attenuation through spatially tailored impedance distributions, synergizing material gradient design and intelligent algorithm optimization to overcome inherent performance limits of conventional absorbers. Key mechanisms include: impedance-gradient layers for broadband matching, complementary spectral response in magnetic/dielectric composites enhancing dissipation, and multi-layer graded architectures extending wave propagation paths. As shown in Figure 7a, Shan et al. [67] engineered quartz fiber/nanotube gradient composites achieving 1.26–25.8 GHz ultra-broadband absorption with mechanical stability. As shown in Figure 7b, Zhang et al. [68] fabricated 3D-printed Kelvin honeycombs attaining 2–40 GHz bandwidth (>89.2% coverage) at 3.75 MPa compressive strength, As shown in Figure 7c, while Dong et al. [69] achieved 6.6 GHz bandwidth with −40.9 dB peak absorption using whale-algorithm-optimized plate-lattice structures. As shown in Figure 7d, Huang et al. [70] demonstrated 91.84% absorption across 2–40 GHz and 2.87 KN load capacity with short carbon fiber-reinforced gradients, exemplifying ultra-broadband/high-toughness synergy. As shown in Figure 7e, Fan et al. [71] validated additive manufacturing capabilities through 5 mm thin-layer structures achieving 2–40 GHz absorption.

Despite challenges in cost-effective manufacturing, gradient structures’ design flexibility and functional scalability continue advancing EM stealth toward ultra-broadband, ultra-thin intelligent systems, providing innovative solutions for next-generation stealth vehicles and UAVs.

#### 3.2.3. Sandwich Structures

**Figure 8:**
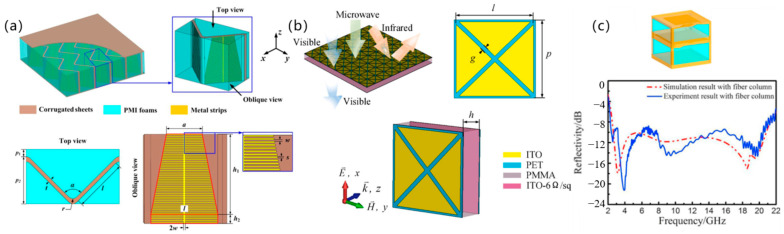
Sandwich structures: (**a**) Lightning-protective highly conductive thin-film sandwich; (**b**) Fiber-column reinforced broadband absorbing structure; (**c**) Optically transparent ITO sandwich architecture [72,73,74]. Reproduced with permission from Composite Structures and Results in Physics and Infrared Physics & Technology.

Sandwich configurations provide an ideal platform for multifunctional integration, achieving synergistic optimization of EM properties, mechanical performance, and environmental adaptability through innovative core designs and composite material engineering. Current research leverages additive manufacturing for precision fabrication, exemplified by laser powder bed fusion (LPBF) processed metallic sandwich structures exhibiting simultaneous lightweight and high-toughness characteristics. As shown in Figure 8a, Jang et al. [72] engineered highly conductive thin-film sandwiches achieving >80% absorption at 5.74–7.84 GHz with integrated lightning-strike protection, whereas their corrugated channel design enables switchable absorption transmission functionality (8.96–12.45 GHz, >80% absorption). As shown in Figure 8b, Shen et al. [73] developed fiber-column reinforced structures attaining 2.6–21 GHz broadband absorption (RL ≤ −10 dB) at 9.73 mm thickness via double-layer resistive films, overcoming traditional absorbers’ mechanical limitations. As shown in Figure 8c, Xu et al. [74] developed an indium tin oxide (ITO) transparent sandwich structure maintaining >90% absorption in 5.8–8.3 GHz with concurrent preservation of optical transparency, advancing optoelectronic integrated stealth technology.

Core tuning mechanisms encompass: graded-index core layers enabling broadband matching, functional cladding layers providing multiphysics protection, and reinforced fiber-column/corrugated structures enhancing mechanical integrity. Despite their modular advantages, sandwich structures exhibit structure-dependent practical limitations: foam-core sandwiches, for instance, are cost-effective but suffer from poor thermal stability (RL variation > 3 dB at 150 °C), making them unsuitable for engine bay applications; fiber-column reinforced cores, while thermally stable, require precise alignment during fabrication—limiting large-area production yields to <60%. Currently, no comparative benchmarks exist for evaluating sandwich structures across critical metrics (e.g., thermal-EM synergy, fabrication yield), hindering selection for specific scenarios (e.g., aerospace vs. high-speed trains). Future work should establish such benchmarks to standardize performance trade-off analysis.

The comparative analysis of EM wave-absorption performance across distinct structural archetypes of composite layered architectures discussed in the preceding section is systematically compiled in Table 4, which details key absorption metrics including effective bandwidth, reflection loss minima, and frequency-specific attenuation characteristics.

### 3.3. Conformal Structures

**Figure 9:**
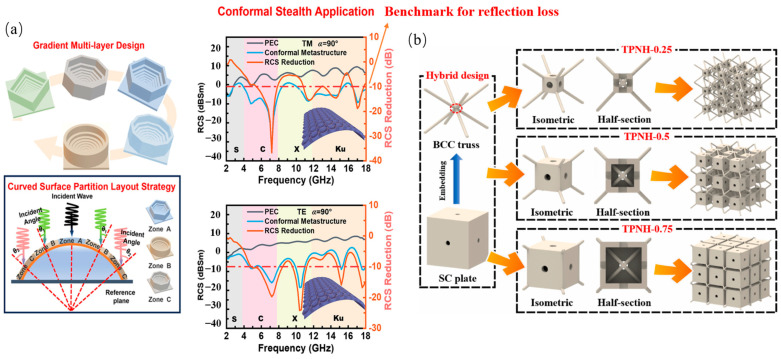
Conformal structures: (**a**) Gradient cell-surface conformal structure; (**b**) Windowed conformal structure [76,77]. Reproduced with permission from the Thin-Walled Structures and Composites Part B: Engineering.

Conformal structures overcome traditional planar configuration limitations through surface adaptive design, exhibiting unique advantages in stealth-protection integration for complex curved targets [75]. As shown in Figure 9a, Chen et al. [76] engineered a gradient meta-atom partitioned structure achieving polarization insensitive broadband absorption (5.4–18 GHz, avg. RL ≤ −10 dB) under high-curvature conditions (90° central angle), revealing curvature-compensation mechanisms via zoned surface layouts. As shown in Figure 9b, Li et al. [77] achieved 80% dual-band absorption (4.5–18 GHz) through a windowed conformal architecture while enhancing blast resistance via multi-window stress dispersion, and demonstrated controlled surface-ablation failure modes (scaled distance 0.646–2.565 m/kg^1/3^) in ballistic testing.

Performance advantages originate from: asymmetric non-periodic meta-atom arrangements suppressing EM fluctuations; reinforcement/window designs optimizing mechanical load distribution; and compliant substrates ensuring surface conformality. These enable stealth-protection integration for fighter aircraft skins and naval vessel superstructures.

Future research will develop dynamic curvature adaptation via shape memory alloys; establish electromagnetic-mechanical-impact co-design methodologies; and advance surface-conformal additive manufacturing. Despite challenges in extreme curvature tolerance and environmental durability, these architectures’ surface compatibility and multifunctional integration will expand EM stealth applications for complex curved platforms.

The study of EM absorption characteristics of the above mentioned composite layered structures of different structural types is summarized as shown in Table 5.

### 3.4. Bio-Inspired Structure

**Figure 10:**
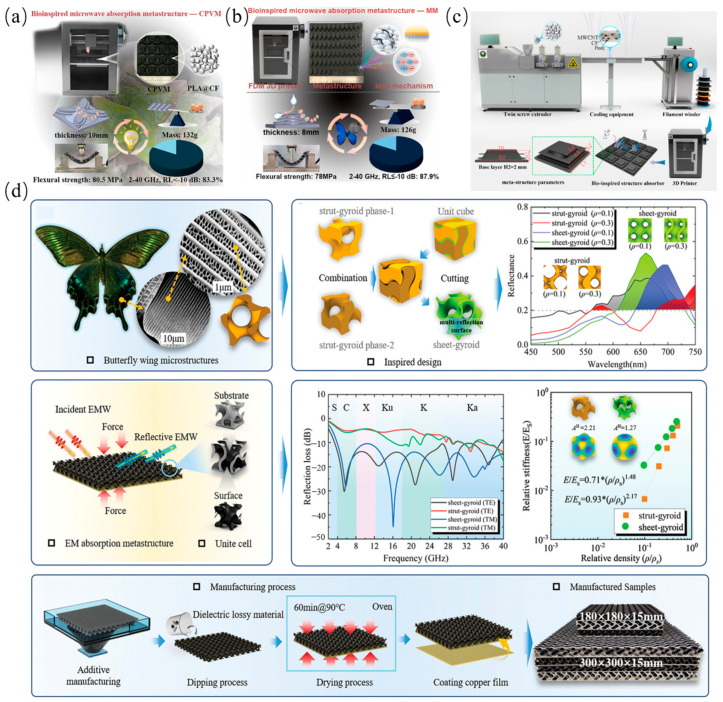
Bionic Structures: (**a**) Butterfly scale structure; (**b**) Plant-inspired structures; (**c**) Topology inspired by succulents; (**d**) Butterfly wing bionic [78,79,80,81]. Reproduced with permission from the Composites Science and Technology and Composites Part B: Engineering and Chemical Engineering Journal and Advanced Materials.

Bio-inspired metamaterials derive design principles from biological systems’ intricate architectures, such as plant vasculature and lepidopteran scales. Researchers achieve synergistic electromagnetic-mechanical optimization by emulating these natural blueprints with intelligent algorithms and advanced fabrication. Such emulation yields core advantages: bio-inspired geometric multiscale impedance matching, helical topologies that extend wave propagation paths, and hierarchical arrangements that enable multi-mechanism loss synergy. Additive manufacturing facilitates complex bio-topology realization, as shown in Figure 10a, Ge et al. [78] demonstrated through their 3D-printed PA6@CF butterfly-scale structure that maintained broadband absorption with superior surface conformality. As shown in Figure 10b, Dong et al. [79] attained 11.5 GHz bandwidth (2–18 GHz) using an arborescent fractal design, while achieving 2–40 GHz coverage (83.3% fractional bandwidth) with succulent-inspired topologies exhibiting 41–80.5 MPa compressive strength [80]. As shown in Figure 10c,d, An et al. [81] induced spherical current vortices and multiscale scattering via lepidopteran-inspired helix microstructures, yielding ≤−10 dB reflection loss across 2.3–40 GHz at 60 incidence alongside 78 MPa flexural strength and 0.132 drag coefficient, thereby providing a feasible approach to integrating EM stealth with aerodynamic performance.

Future research will focus on environmentally adaptive systems using stimulus-responsive materials; multiscale optimization via deep learning based bio-structure-performance mapping; and extreme environment applications (e.g., deep-sea/space multifunctional integration). Despite their structure-function advantages, bio-inspired metamaterials face pronounced practical limitations tied to their topological complexity: butterfly-scale structures, for example, achieve ultra-broadband absorption (2.3–40 GHz) but require nanoscale precision fabrication—resulting in production costs ~5× higher than conventional laminates; succulent-inspired gradient structures, while mechanically resilient, suffer from inaccurate EM loss prediction due to irregular pore distribution. Notably, no comparative benchmarks currently exist for evaluating ‘fabrication complexity vs. absorption performance’ across bio-inspired archetypes, complicating material selection for submersible or aerospace applications. Future research must address this gap to prioritize low-complexity, high-performance designs.

The studies on the EM absorption characteristics of the above mentioned bionic structures of different structural types are summarized as shown in Table 6.

### 3.5. Metasurfaces

**Figure 11:**
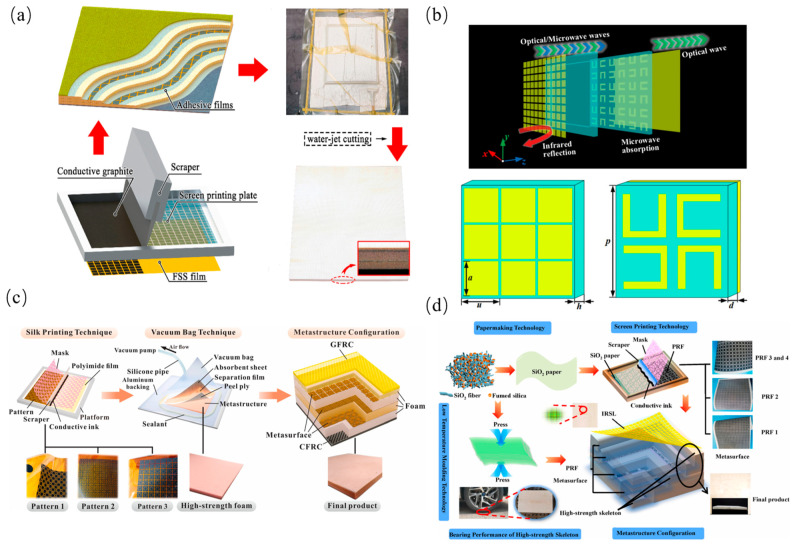
Metasurfaces: (**a**) Frequency-selective surface (FSS) based metasurface; (**b**) ITO-based transparent metasurface; (**c**) Patterned resistive film; (**d**) Silica fiber metasurface [82,83,84,85]. Reproduced with permission from the Composites Science and Technology and Infrared Physics & Technology and Composites Science and Technology and Composites Part B: Engineering.

Metasurfaces enable multidimensional EM wave manipulation through subwavelength unit engineering, with significant progress reported in multifunctional absorption and creating new pathways for multispectral compatibility. Current research automates complex metasurface design via intelligent algorithms, exemplified by Wang et al.’s frequency-selective surface (FSS) architecture combining GFRP, poly vinyl chloride (PVC) foam, and artificial loss films for customizable absorption with structural load-bearing [82]. As shown in Figure 11a, Yuan et al. [83] developed ITO transparent metasurfaces achieving 4–18 GHz absorption with 76% visible transmittance and <0.24 infrared emissivity, setting multispectral stealth records. As shown in Figure 11b, Huang et al. [84] attained 2.0–22.9 GHz ultra-wideband absorption at 16.05 mm thickness through patterned resistive films and genetic algorithm optimization, maintaining 23.12 MPa flexural strength as shown in Figure 11c,d, while An et al.’s silica fiber metasurfaces preserved 3.1 GHz bandwidth at 1000 °C, enabling extreme-environment operation [85].

These advances originate from: localized EM resonance enhancing energy dissipation, periodic dispersion engineering extending propagation paths through coherent scattering, and multiphysics functional coupling in multimaterial systems. Future research will develop dynamically reconfigurable systems using phase-change materials for real-time spectral control; implement multiphysics digital twins for electromagnetic-mechanical-thermal behavior prediction; and integrate smart materials like self-healing coatings for enhanced durability. Despite their tunable EM control, metasurfaces exhibit structure-specific practical barriers: ITO-based transparent metasurfaces, while suitable for optoelectronic-stealth integration, have limited infrared compatibility (emissivity >0.24), restricting their use in multispectral stealth; patterned resistive film metasurfaces, though achieving ultra-wideband absorption (2.0–22.9 GHz), require photolithography with <5 μm precision—limiting large-area production to <1 m^2^ per batch. Furthermore, no cross-structural comparative benchmarks exist (e.g., metasurfaces vs. Three-Dimensional lattices for weight-sensitive aerospace applications), leading to inconsistent performance claims. Future work must establish such benchmarks to enable objective evaluation of metasurfaces against other MF-MM architectures.

The study of EM absorption properties of different structural types of hypersurfaces mentioned above is summarized as shown in Table 7.

### 3.6. Composite Metamaterials

Composite metamaterials enable targeted EM performance optimization through synergistic material combinations and microstructural engineering, establishing a critical paradigm for wave-absorption applications. These systems overcome conventional absorbers’ functional limitations by balancing broadband absorption, mechanical robustness, and environmental adaptability via compositional-structural co-design.

**Figure 12:**
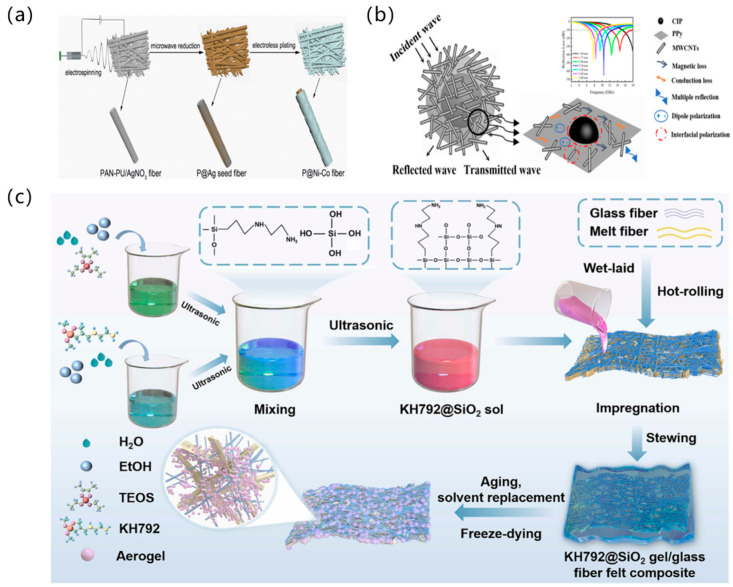
Composite metamaterials: (**a**) Ni-Co nanofiber composites; (**b**) CIP@PPY/MWCNTS composites; (**c**) graphene-polyaniline composites [87,88,89]. Reproduced with permission from the Journal of Alloys and Compounds and Diamond and Related Materials and Construction and Building Materials.

Fan et al. [86] implemented genetic-algorithm-optimized carbon-ink composites achieving >90% absorption across 2–26 GHz with exceptional environmental stability. As shown in Figure 12a, Zhang et al. [87] engineered 0.18 mm Ni-Co nanofiber membranes attaining 77.8 dB average absorption in 8–26.5 GHz, synergizing 1139.6 S/cm conductivity with 49.6 emu/g magnetic saturation for lightweight protection. As shown in Figure 12b, Li et al. [88] demonstrated ternary core–shell structures coordinating carbonyl iron (magnetic loss), polypyrrole (dielectric loss), and MWCNTs (conductive network), achieving 5.80 GHz Ku-band absorption at 1.77 mm thickness. Their nitrogen-doped cobalt@carbon composites maintained 11–18 GHz bandwidth at 10% filling ratio, significantly enhancing material utilization efficiency. As shown in Figure 12c, Xue et al. [89] constructed graphene-polyaniline array-enhanced α-MnO_2_ nanostructures attaining 7.5–13.7 GHz absorption with −36.5 dB peak loss. Choi et al. [90] balanced mechanical-electromagnetic properties in SiC fiber-reinforced composites, enabling aerospace skin and EMI shielding applications.

Future research will: develop stimulus-responsive systems for dynamic absorption switching; establish cross-scale multiphysics models predicting electromagnetic-thermal-mechanical couplings; and innovate green manufacturing processes for industrial scalability. Composite metamaterials continue advancing EM stealth toward ultra-wideband intelligent multifunctionality, providing design strategies for next-generation defense-related EM protection.

The studies on the EM absorption properties of the different composites mentioned above are summarized as shown in Table 8.

## 4. Conclusions and Outlook

### 4.1. Conclusions

This review systematically analyzes advancements in MF-MMs for EM wave absorption, focusing on “structure-material-function” co-design strategies to overcome traditional absorbers limitations of narrow bandwidth, functional singularity, and poor environmental adaptability.

Structural innovations drive MF-MM performance, but each archetype exhibits distinct practical boundaries: 3D lattice structures excel in mechanical-EM synergy (load-bearing capacity up to 935% higher than conventional absorbers) but are impractical for dynamic EM environments due to fixed impedance; composite laminates achieve ultra-broadband absorption (1.26–40 GHz) and ultrathin profiles (<5 mm) yet suffer from delamination risks under cyclic thermal stress; bio-inspired topologies offer wide-angle absorption but lack scalable fabrication methods. Critically, the field currently lacks standardized comparative benchmarks for key metrics (e.g., bandwidth-thickness ratio, extreme temperature stability, fabrication cost), which are essential to unify performance evaluation and accelerate practical adoption. Synergistic impedance matching and EM dissipation mechanisms are critical for enabling efficient absorption. Optimizing complex permittivity (*ε*) and permeability (*μ*), coupled with rational structural engineering, enhances dielectric/magnetic losses and multi-resonance effects, maximizing EM wave penetration and energy dissipation.

MF-MMs demonstrate transformative potential in resolving broadband stealth and mechanical load-bearing integration challenges, providing innovative solutions and technical foundations for EM protection in defense and aerospace platforms.

### 4.2. Outlook

Despite considerable progress, significant challenges remain in the practical application of multifunctional metamaterial absorbers. Future research should prioritize: (1) developing intelligent, adaptive systems using stimuli-responsive materials (e.g., shape memory alloys, phase-change polymers) integrated with flexible electronics for real-time absorption tuning in dynamic EM environments; (2) establishing structure-specific multiphysics modeling frameworks: for 3D lattice structures, prioritize coupling EM parameters with dynamic mechanical deformation to address tunability gaps; for bio-inspired topologies, integrate fabrication-induced defects into thermal-EM models to improve scalability predictions; for metasurfaces, develop coupled thermal-resistive models to resolve incomplete multiphysics coupling. These frameworks should further enable the establishment of comparative benchmarks (e.g., EM absorption retention under 1000 cyclic loads, bandwidth stability at −50 to 150 °C) to standardize performance evaluation across all MF-MM structural archetypes; (3) enhancing stability in extreme environments through advanced matrix materials (ceramic-matrix composites, intermetallic compounds) and degradation-resistant topological designs to enable aerospace and deep-sea deployment; and (4) accelerating translation to industrial applications via additive manufacturing (laser powder bed fusion) combined with heuristic optimization algorithms (genetic algorithms, whale optimization) for cost-effective, high-precision fabrication of complex metastructures with proven long-term reliability.

In summary, research on EM wave-absorbing MF-MMs will advance toward adaptive intelligence, multifunctional integration, and scalable engineering. These developments are expected to yield improved material systems and design frameworks for next-generation EM shielding and low-observability platforms.

## Figures and Tables

**Table 1:** Key Performance Metrics for Metamaterial Electromagnetic Wave Absorbers.

Performance Metric	Core Significance
Absorption Strength	Characterized by a reflection loss (RL) ≤ −10 dB, corresponding to ≥90% absorption efficiency.
Absorption Bandwidth	Defined as the frequency range over which RL ≤ −10 dB is maintained.
Thickness & Weight	Thickness: Typically ≤ 50 mm (for practical applications) Areal Density: Typically ≤ 1.5 g/cm^3^ (for practical applications)
Thermal Stability	Variation in RL (ΔRL) ≤ 2 dB over the operational temperature range (−50 °C to 150 °C)

**Table 2:** Structural Classification of Multifunctional Metamaterials.

Structural Type	Primary Mechanism	Absorption Characteristics	Representative Applications
3D Lattice Structures	Multi-reflection & Gradient Impedance	Broadband/High-Loss	Aerospace Stealth
Composite Stratification	Interference-Dissipation Synergy	Broadband/Multi-Mechanism	High-Temperature EM Absorption
Conformal Architectures	Multi-Scale Resonance & Scattering	Broadband	Wideband Absorbing Materials
Bioinspired Topologies	Anti-Reflection & Multi-Level Loss	Wide-Angle/Broadband	Infrared-Microwave Compatible Stealth
Metasurfaces	Phase Discontinuity & Near-Field Enhancement	Narrowband/Tunable	Flexible Stealth Screens

**Table 3:** Absorption Performance Metrics of 3D Lattice Structures.

Structural Archetype	Effective Bandwidth (GHz)	Min. RL (dB)	Thickness/Unit Cell Size (mm)	Figure	Reference
Honeycomb	3–16	−35	0.2	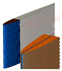	[47]
	2–20.37	−41.6	4.1	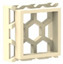	[49]
	2.7–25.2	−17	0.1	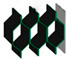	[50]
	1.92–17.6	−30	---	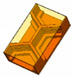	[51]
Truss	7–17	−58.2	1.2	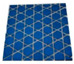	[53]
	5.8–18	−30.7	1.2	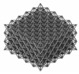	[54]
	2–6.8 10.4–40	---	4	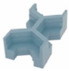	[55]
Other Periodic Lattice	5.4–18	---	0.2	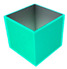	[58]
	3.42–19.73	−34	3.5	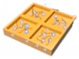	[59]
	12.87–17.78	−29.2	1.62	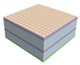	[60]
	5.8–18	−24	3.4	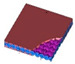	[61]
	8.7–19.2	−30.5	2.7	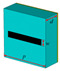	[62]

**Table 4:** Absorption Performance Metrics of Composite layered structures.

Structural Archetype	Effective Bandwidth (GHz)	Min. RL (dB)	Thickness/Unit Cell Size (mm)	Figure	Reference
Multilayer	8.2–12.4	−35	2.37	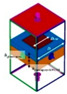	[63]
	4.7–13.7	−22.3	2.5	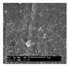	[64]
	8.2–12.4	−33.5	3.9	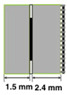	[65]
	8.2–12.4	−32.3	1.84	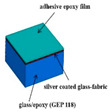	[66]
Gradient	1.26–25.8	−25	9.1	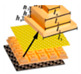	[67]
	3.03–18	−26	21	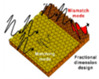	[68]
	5.3–39.8	−15	2.2	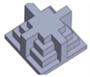	[69]
	2–18 26.6–40	−15	5	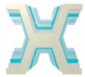	[70]
	5.25–39.47	−39.95	2	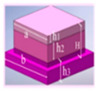	[71]
Sandwich	5.74–7.84	−19	0.2	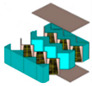	[72]
	2.6–21	−21	9.73	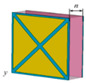	[73]
	5.8–8.3	−18	3.5	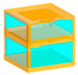	[74]

**Table 5:** Absorption Performance Metrics of Conformal structures.

Structural Archetype	Effective Bandwidth (GHz)	Min. RL (dB)	Thickness/Unit Cell Size (mm)	Figure	Reference
Conformal	5.4–18	−22.3	30	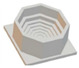	[76]
	4.5–18	---	20	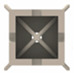	[77]

**Table 6:** Absorption Performance Metrics of Bionic structures.

Structural Archetype	Effective Bandwidth (GHz)	Min. RL (dB)	Thickness/Unit Cell Size (mm)	Figure	Reference
Bio-inspired	2.9–18 23–40	−47.7	8	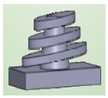	[78]
	4–15.5 18–36.2	−40	10.8	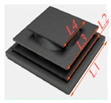	[79]
	4.44–40	−54.4	13	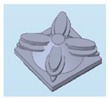	[80]
	2.3–40	---	15	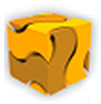	[81]

**Table 7:** Absorption Performance Metrics of Metasurfaces.

Structural Archetype	Effective Bandwidth (GHz)	Min. RL (dB)	Thickness/Unit Cell Size (mm)	Figure	Reference
Metasurfaces	2–22	---	17.4	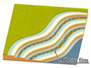	[82]
	4–18	---	3	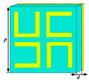	[83]
	2–22.9	−36.7	16.05	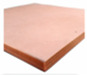	[84]
	2–3.5 5.4–18	−22.3	8.6	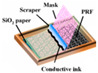	[85]

**Table 8:** Electromagnetic Wave Absorption Properties of Selected Composite Metamaterials.

Materials System	Effective Bandwidth (GHz)	Thickness/Unit Cell Size (mm)	SEM Figure	Reference
Peek/CB	2–26	---	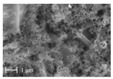	[86]
PAN-PU-AgNO_3_	8–26.5	0.18	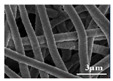	[87]
CIP@PPY/MWCNTS	12.2–18	1.77	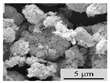	[88]
KH792/SiO_2_	7.5–13.7	10	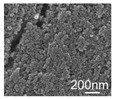	[89]
SiC/epoxy	8–12	0.24	---	[90]

## Data Availability

No new data were created or analyzed in this study. Data sharing is not applicable to this article.
